# Augmented Risk Prediction for Alzheimer’s Onset From Electronic Health Records With Large Language Models

**DOI:** 10.1093/geroni/igaf122.1207

**Published:** 2025-12-31

**Authors:** Jiankun Wang, Sumyeong Ahn, Taykhoom Dalal, Xiaodan Zhang, Bin Chen, Hiroko Dodge, Fei Wang, Jiayu Zhou

**Affiliations:** University of Michigan, Ann Arbor, Michigan, United States; Michigan State University, East Lansing, Michigan, United States; Weill Cornell Medicine, New York, New York, United States; Michigan State University, East Lansing, Michigan, United States; Michigan State University, East Lansing, Michigan, United States; Massachusetts General Hospital, Harvard Medical School, Boston, Massachusetts, United States; Weill Cornell Medicine, New York, New York, United States; University of Michigan, Ann Arbor, Michigan, United States

## Abstract

**Background:**

Alzheimer’s disease and related dementias (ADRD) are among the top causes of mortality and disability in older adults. Early detection is critical for timely clinical intervention, caregiver planning, and patient enrollment in clinical trials. Electronic health records (EHRs) offer a readily accessible data source for automated dementia risk screening. However, existing predictive models often struggle to accurately identify nuanced or complex patient presentations, limiting their clinical utility. Recently, large language models (LLMs)—advanced artificial intelligence systems proficient at reasoning and interpreting medical data—have demonstrated promising capabilities for improving diagnostic accuracy.

**Methods:**

We propose a novel dementia risk prediction pipeline that integrates traditional supervised machine learning methods (SLs) with advanced LLMs. Our method uses SLs to accurately classify clear-cut dementia risk cases from structured EHR data, while leveraging LLMs’ sophisticated reasoning capabilities to handle ambiguous or challenging patient records. We evaluated this collaborative approach using a large, real-world EHR dataset from Oregon Health & Science University (OHSU), consisting of over 2.5 million patient records spanning more than 20 million clinical encounters.

**Results:**

Our integrated approach significantly outperformed traditional predictive models alone, demonstrating improved accuracy and reliability in dementia risk prediction. The combined use of SLs and LLMs effectively captured subtle clinical indicators that traditional methods overlooked.

**Conclusion:**

Integrating advanced language models into existing machine learning approaches markedly improves dementia screening from routine clinical data. This method shows substantial potential for enhancing early ADRD detection, offering physicians powerful tools for proactive, personalized patient management.

